# Case Report: Blurred Vision and Eruptive Nevi - Bilateral Diffuse Uveal Melanocytic Proliferation With Mucocutaneous Involvement in a Lung Cancer Patient

**DOI:** 10.3389/fonc.2021.658407

**Published:** 2021-04-13

**Authors:** David Rafei-Shamsabadi, Johanna Schneider, Laura Trefzer, Kristin Technau-Hafsi, Frank Meiss, Thomas Ness

**Affiliations:** ^1^ Department of Dermatology and Venereology, Medical Center - University of Freiburg, Faculty of Medicine, University of Freiburg, Freiburg, Germany; ^2^ Department of Medicine IV, Medical Center - University of Freiburg, Faculty of Medicine, University of Freiburg, Freiburg, Germany; ^3^ Eye Center, University Hospital Freiburg, Faculty of Medicine, University of Freiburg, Freiburg, Germany

**Keywords:** bilateral diffuse uveal melanocytic proliferation (BDUMP), skin involvement, melanoma, plasmapheresis, non-small-cell lung cancer (NSCLC)

## Abstract

We describe a case of a 65-year old patient presenting with unusual mucocutaneous melanocytic proliferations of a Bilateral Diffuse Uveal Melanocytic Proliferation (BDUMP) imitating a multifocal melanoma in situ, which improved dramatically after plasmapheresis. The patient first presented at the dermatology department due to rapidly evolving brown and black macules on the glans penis. Further skin involvement of the perineal and perianal region, mamillae and oral mucosa was stated. Histology from a penile biopsy was compatible with a melanoma in situ. Due to the distribution pattern and elevated serum tumor marker S100B, metastatic melanoma was considered. Staging examinations using PET-CT scan however, revealed a lung tumor, later confirmed as a Non-small-cell lung cancer (NSCLC). Primary radio chemotherapy was initiated to treat NSCLC. Shortly after initiation of radio chemotherapy the patient developed massive vision impairment and a NSCLC-associated BDUMP was diagnosed which led to the correct classification of melanocytic skin lesions as mucocutaneous BDUMP manifestation. Plasmapheresis was started resulting in a rapid improvement of vision starting ten days after the first plasmapheresis. In contrast skin manifestations started to disappear with a marked delay 4 months after the last plasmapheresis cycle. This case highlights the importance of memorizing multiple rapidly progressing melanocytic skin and/or mucous membrane spots together with visual impairment as a possible paraneoplastic BDUMP that needs a fundamentally different therapeutic approach compared to multifocal melanoma in situ.

What is already known about this topic? Bilateral Diffuse Uveal Melanocytic Proliferation (BDUMP) is a paraneoplastic syndrome with melanocytic uveal proliferation leading to vision impairment. Extraocular manifestation is rare, mainly affect the subepidermal compartment and is hard to treat. Plasmapheresis has been shown to be an effective treatment mainly for vision improvement in some but not all cases.

What does this study add? Our BDUMP case with widespread skin and mucosal involvement initially mimicked a multifocal melanoma *in situ* and showed an excellent treatment response to plasmapheresis. Improvement of mucocutaneous lesions has not been documented well in the literature so far. We show a more than one year lasting follow up still underlining the beneficial effect of plasmapheresis in this case. In-vitro data supports the hypothesis that plasma exchange eliminates a “Cultured melanocyte elongation and proliferation (CMEP)” factor out of patient blood leading to decreased melanocyte proliferation shown numerically in-vitro and clinically in-vivo. Our case clearly indicates that before establishing a definite diagnosis and therapy in patients with rapidly evolving melanocytic skin and/or mucosal lesions BDUMP mimicking multifocal melanoma *in situ* should be considered making a thorough diagnostic workup mandatory.

## Introduction

We present a case of a Non-small-cell lung cancer (NSCLC)-associated Bilateral Diffuse Uveal Melanocytic Proliferation (BDUMP), which mimicked a multifocal melanoma *in situ* with diffuse skin and mucous membrane involvement showing epidermal proliferation only. Impaired vision dramatically improved shortly after start of plasmapheresis treatment whereas mucocutaneous lesions showed regression with profoundly delayed dynamics. The patient gave written consent for publishing clinical pictures and data gained from scientific usage of skin biopsies and plasma.

## Patient Information

**Figure 1:**
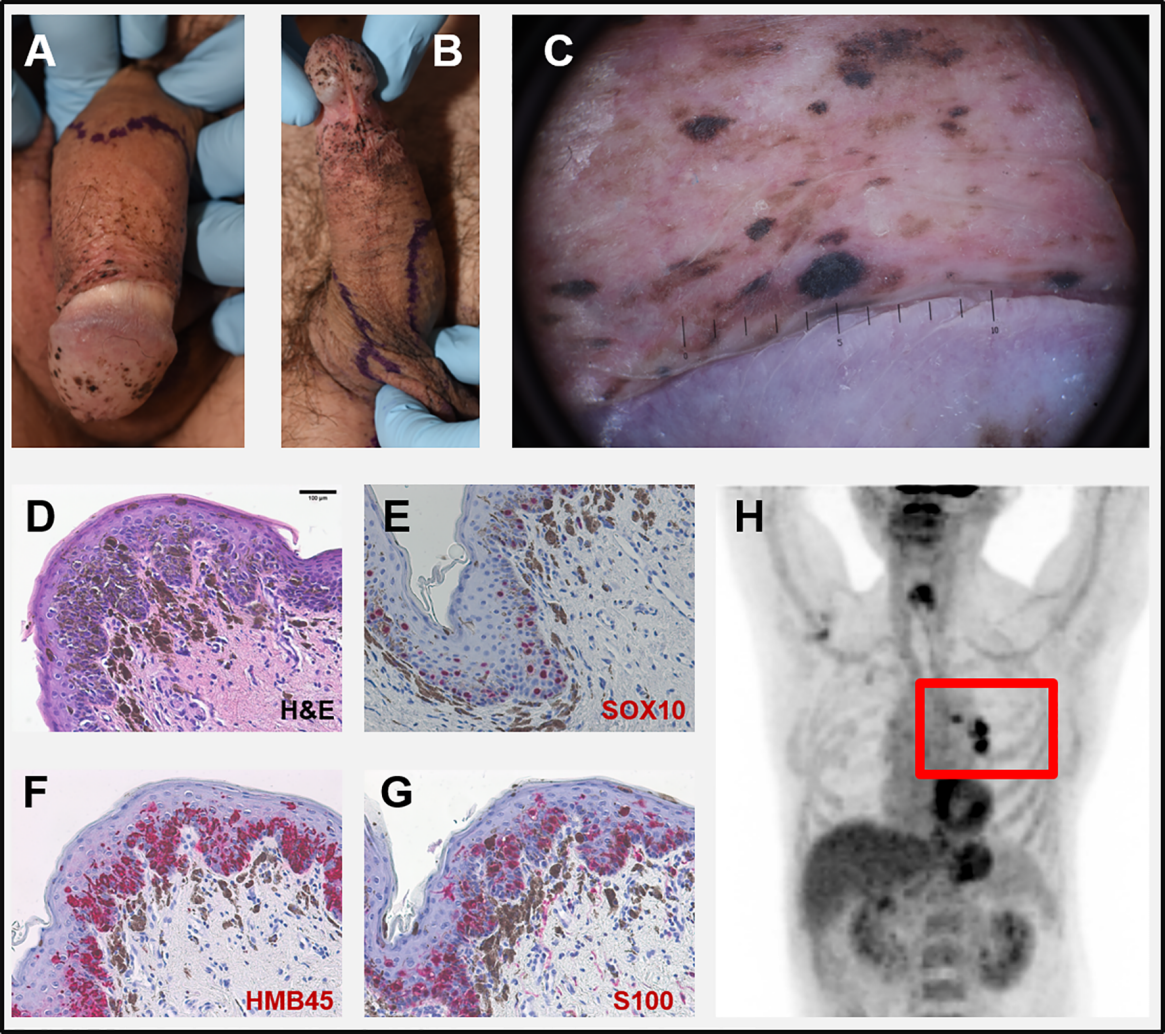
**(A, B)** Clinical pictures at first visit: multiple slightly palpable black spots on penis and scrotum. **(C)** Dermoscopy of a corresponding lesion of the glans penis: homogeneous dark-brown and black spots largely lacking net-like structures. **(D–G)** Microscopic pictures of a biopsy taken from the penis. (**D)** H&E-staining showing atypical melanocytes with focal pagetoid infiltration of the epidermis and strong junctional melanocytic pigmentation with pigmentary incontinence. Intraepidermal melanocytes strongly express SOX10 **(E)**, HMB45 **(F)**, and S100 **(G)**. **(H)** PET-CT scan with highly suspicious lymph nodes in the left hilus region (red rectangle). Scale bar in D = 100 µm.

A 65-year old male patient was sent to our outpatient department with rapidly growing black macules on the penis (**Figures 1A, B**). He reported no clinical symptoms at that time but was concerned about the increasing extent of these spots. The patient had a history of larynx carcinoma with neck dissection and adjuvant radiotherapy and squamous cell carcinoma of the left conjunctiva also excised with clear margins. Both tumors occurred more than 10 years ago. The patient had smoked for several years but had quit 12 years prior. The family history was unremarkable concerning malignant diseases.

## Clinical Findings

At first visit, multiple black, homogenous spots some of them discretely sublime on the glans penis, dorsum penis and scrotum were evident (**Figures 1A, B**). Dermoscopy of these lesions showed homogeneous dark-brown and black spots largely lacking net-like structures (**Figure 1C**). No other pathologic skin lesions were identified. No pathological lymph nodes were palpable.

## Diagnostic Assessment

Histology of biopsies from penis and scrotum revealed multifocal melanoma *in situ* with atypical melanocytes showing a focal pagetoid infiltration of the epidermis and strong junctional melanin pigmentation with marked pigmentary incontinence (**Figure 1D**). Intraepidermal melanocytes expressed SOX10, HMB45, und S100 (**Figures 1E–G**). The serum S100B level, a tumor marker for malignant melanoma, was increased (0.353 µg/l, norm <0.105 µg/l) while lactate dehydrogenase (LDH) was normal. The considered diagnosis was a metastatic melanoma with multiple skin lesions (epidermotropic metastases) with unknown primary. A PET-CT scan revealed a suspicious lesion in the lower lobe of the left lung and pathological lymph nodes in the corresponding hilus region (**Figure 1H**). No further suspicious lesions were detected. An MRI of the brain was normal. Endobronchial ultrasound (EBUS) guided biopsies detected a pleomorphic adenocarcinoma of the lung (NSCLC) but no signs of malignant melanoma.

## Therapeutic Intervention

**Figure 2:**
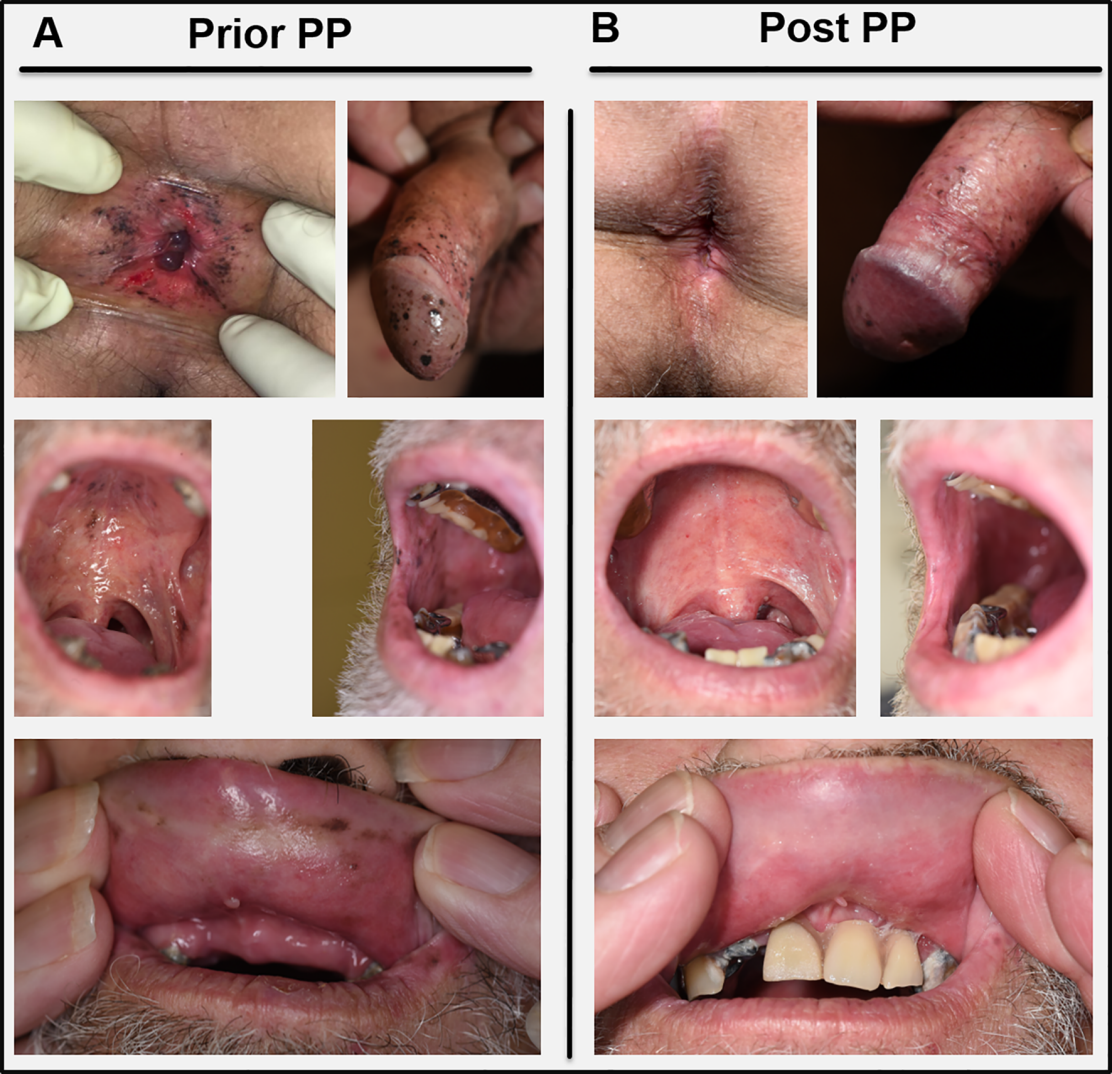
**(A)** Skin and mucous membrane manifestations of BDUMP before initiation of plasmapheresis (Prior PP). **(B)** Clinical presentation four months after the last PP cycle (Post PP) with profound decrease of melanocytic proliferations especially on the oral mucous membranes and in the perianal region. Pictures in the lowest panel were taken without (left) and with (right) the patients’ dental prosthesis.

**Figure 3:**
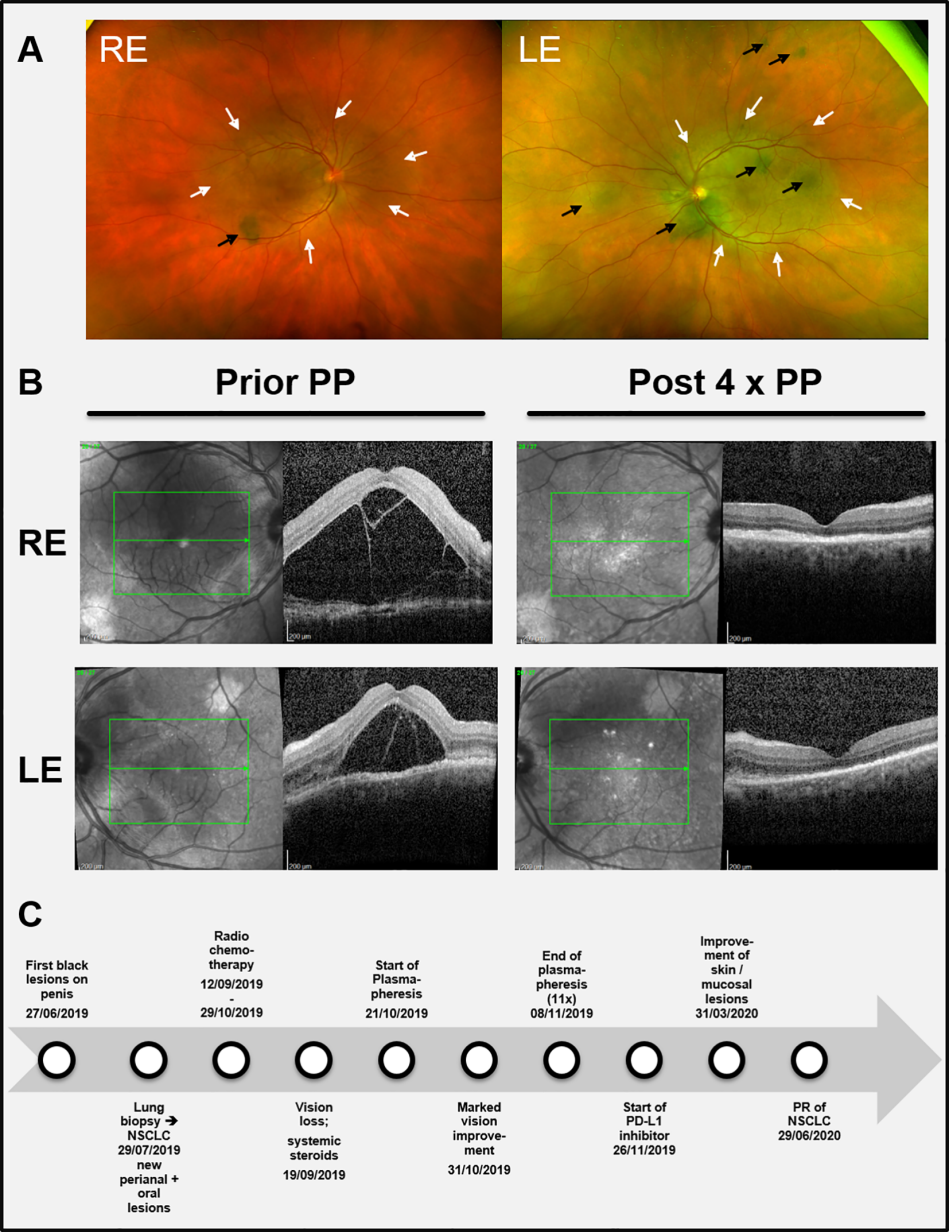
**(A)** Color funduscopic pictures showing the initial ocular findings. Black arrows: subretinal melanocytic proliferations; white arrows: area of serous retinal detachment. **(B)** (left part): Black and white funduscopic and optical coherence tomography (OCT) images of the right eye (RE) and the left eye (LE) before start of plasmapheresis (Prior PP) when visual impairment was most prominent leading to total blindness. (**B** right part) Corresponding images after four cycles of plasmapheresis (Post 4 x PP) showing a remarkable reduction of the subretinal edema and the retinal detachment, respectively. **(C)** Timeline summarizing the relevant data from the episode of care. NSCLC = Non-small-cell lung cancer. PD-L1 = Programmed death-ligand 1.

Under the initial assumption of two malignancies, we first planned a curative resection of the multifocal melanoma *in situ* followed by an operation and subsequent adjuvant therapy of the NSCLC. Meanwhile the patient had developed further melanocytic lesions on the oral mucosa and the perianal region (**Figure 2A**). Thus, resection was abandoned. Instead, primary radio chemotherapy (cumulative dose of 66 Gy + Cisplatin and Vinorelbine) of the NSCLC was started. During radio-chemotherapy, the patient experienced a sudden impairment of vision on both eyes with a complete vision loss. Funduscopic and optical coherence tomography (OCT) showed subretinal melanoma like lesions with a massive serous retinal detachment in both eyes (**Figures 3A, B**). Based on these findings paraneoplastic Bilateral Diffuse Uveal Melanocytic Proliferation (BDUMP) was diagnosed and pigmentary skin lesions were reevaluated as mucocutaneous manifestation of BDUMP. Initially, high dose systemic steroids for one week did not lead to any improvement of vision and thus plasmapheresis (11 times over a period of 3 weeks with exchange of one plasma volume per procedure) was started.

## Follow-up and Outcomes

After seven cycles of plasmapheresis cycles (10th day after start of treatment), the patient experienced a rapid improvement of vision. Subsequently, retinal detachment declined as well and vision turned back to normal (**Figure 3B**). The melanocytic skin and mucous membrane lesions however initially remained stable. Four months after the last plasmapheresis cycle melanocytic lesions started to decrease as well (**Figure 2B**). The NSCLC showed stable disease under radio chemotherapy and therapy was switched to a PD-L1-inhibitor (Durvalumab) resulting in a partial response (for a timeline summarizing the relevant data from the episode of care see **Figure 3C**).

## *In-vitro* data

**Figure 4:**
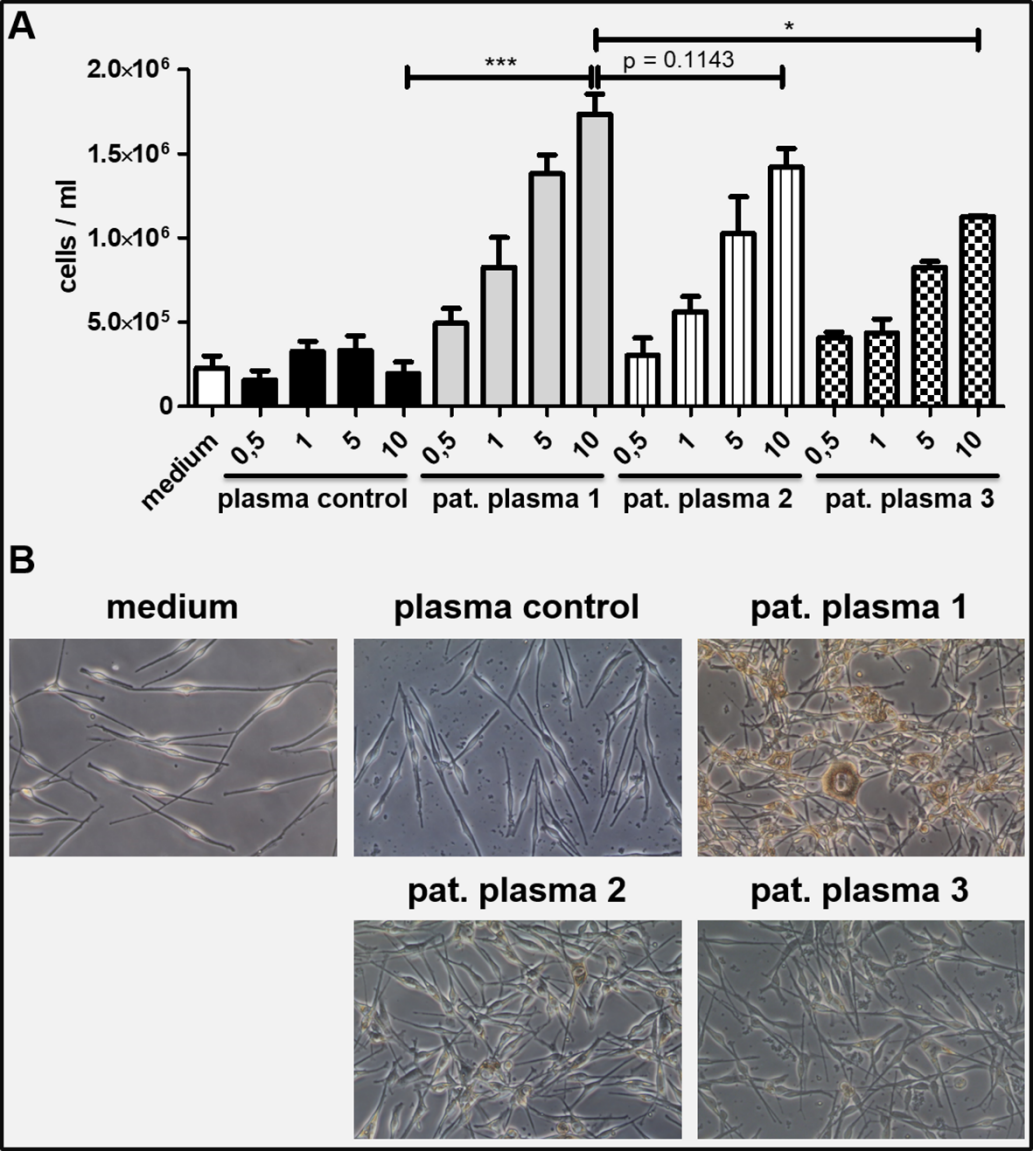
**(A)** Neonatal human melanocytes were incubated with patient plasma under daily medium changes for 7 days. A concentration dependent increase of total cell numbers was observed which was not seen with healthy control plasma and medium control. The effect diminished with ongoing plasmapheresis (1 = plasma from first plasmapheresis; 2 = plasma from second plasmapheresis; 3 = plasma taken 4 months after last plasmapheresis; x-axis showing percentage of plasma volume diluted in culture medium. **(B)** Microscopic pictures of the melanocyte cultures on day 7 (10% plasma conditions): Melanocytes incubated with 10% patient plasma 1 show markedly increased proliferation, more dendrites and higher melanin production compared to plasma control and to a lesser extent to patient plasma from second plasmapheresis and 4 months after last plasmapheresis, respectively. An unpaired, two-tailed t test was performed to compare conditions with 10% plasma volume. Conditions were run at least in duplicates. Values are shown as mean ± SD. *P ≤ 0.05, ***P ≤ 0.001.

Patient plasma obtained before and after plasmapheresis was incubated together with neonatal human epidermal melanocytes for one week in-vitro. Compared to healthy control plasma and medium alone, patient plasma lead to a concentration dependent significant increase in melanocyte cell numbers, melanin pigmentation and elongation of dendrites (**Figures 4A, B**). This effect decreased after the fourth cycle of plasmapheresis and further diminished 4 months after the last treatment. However, the number of proliferating melanocytes was still higher compared to the controls (**Figure 4A**).

## Discussion

In the literature, BDUMP is described as a paraneoplastic disease accompanying predominantly tumors of the female urogenital tract and the lung. Mucocutaneous manifestations of BDUMP have been described in around 10-15% of cases (1, 2). As a possible pathogenic factor secreted by tumor cells the so called “Cultured melanocyte elongation and proliferation (CMEP)” factor has been discussed which presumably belongs to the IgG fraction of serum (3). Possible candidates promoting melanocytic growth comprise α‐melanocyte‐stimulating hormone (α‐MSH), adrenocorticotropic hormone (ACTH), fibroblast growth factor (FGF), hepatocyte growth factor (HGF), granulocyte macrophage colony‐stimulating factor (GM-CSF) and endothelins (4, 5).

Therapeutic options for BDUMP besides treatment of the underlying tumor are scarce. Systemic steroids were effective in some cases (1). However, our patient did not experience any improvement of vision after one week of high dose systemic steroids. Plasmapheresis has been shown to reduce the sub-retinal fluid and to improve vision (6–9). This holds true for our case since retinal detachment and loss of vision was completely reversed already after eleven cycles of plasmapheresis.

Pretreatment plasma dramatically increased melanocyte proliferation *in vitro*. Plasmapheresis *via* a column with a pore size of 0.3 µm lead to a fast and long-lasting remission of melanocytic proliferation starting in the uveal area. Furthermore, with the apheresis devices used the IgG fraction of the serum is being reduced significantly as shown by Hafer et al. (10). Thus, it is tempting to speculate that the concentration of CMEP factor has been successfully decreased by this treatment as seen by the clinical benefit and the reduced capacity of the patient plasma to induce melanocyte proliferation *in vitro*. The delayed onset of treatment response of mucocutaneous lesions compared to uveal lesions could be explained by the superior blood supply in the uvea. In contrast to other cases with skin involvement (11), our and other BDUMP cases (5, 12) showed solely intraepidermal melanocytic proliferation where blood vessels are missing and growth factors act *via* diffusion to the epidermal compartment and hence the effect of reducing CMEP by plasmapheresis may be delayed in intraepidermal variants. However, since we could not take samples from all skin and mucosal lesions we therefore cannot fully exclude that dermal proliferation was present at other sites in our patient. According to the hypothesis of Gass et al. (12), the CMEP factor may act on preexisting nevus cells in the dermal and/or epidermal compartment leading to BDUMP with multifocal pigmented lesions.

Although skin lesions including the genital area and mucosal involvement have been described in a few BDUMP cases (2, 5, 11, 13, 14), our case is unique regarding the extent of skin and mucous membrane involvement, the solely epidermal proliferation and the reported response-pattern of these lesions to plasmapheresis. Little is known about the long-term development of the melanocytic lesions under therapy. In our case, plasma exchange showed immediate improvement of vision and a nearly complete reduction of mucocutaneous melanocytic lesions, which continues to last up to one year and started long before treatment response of the underlying NSCLC could be achieved by systemic therapy.

We cannot rule out, that radio chemotherapy and/or immunotherapy of the NSCLC had an additive effect on the improvement of the mucocutaneous BDUMP lesions. The imminent improvement of vision however underlines the anti-proliferative effect of plasmapheresis.

Before establishing a definite diagnosis and therapy in patients with rapidly evolving melanocytic skin and/or mucosal lesions BDUMP mimicking multifocal melanoma *in situ* should be considered. Thorough clinical and radiological diagnostics including fundoscopy should be performed. Finally, plasmapheresis is a well-tolerated treatment option for BDUMP, which can be performed in parallel to systemic tumor treatments or radiotherapy. A long-term follow up is needed if possible to assess the therapeutic effect of plasmapheresis on mucocutaneous BDUMP lesions.

## Patient Perspective

“I was scared when I suddenly lost my vision. I had to trust the physicians to fix that. Receiving this “blood cleaning” while not seeing anything was weird but I was so glad when I could see again after some days. I hope the lung tumor will continue to shrink as well.” (Patients’ quote).

## Data Availability Statement

The raw data supporting the conclusions of this article will be made available by the authors, without undue reservation.

## Ethics Statement

Written informed consent was obtained from the individual(s) for the publication of any potentially identifiable images or data included in this article.

## Author Contributions

DR-S provided the leading contribution in: conceptualization, data curation, formal analysis, investigation, methodology, project administration, resources, software, visualization, writing-original draft, writing-review & editing. JS supported in data curation, investigation, methodology, resources, writing-review & editing. LT supported in data curation, investigation, writing-review & editing. KT-H supported in data curation, investigation, resources, writing-review & editing. FM provided substantial support in conceptualization, investigation, methodology, supervision, writing-original draft, writing-review & editing. TN provided support in conceptualization, data curation, investigation, project administration, resources, visualization, writing-review & editing. All authors contributed to the article and approved the submitted version.

## Funding

DR-S was supported by the clinician scientists program Excellent Clinician Scientists in Freiburg – Education for Leadership (EXCEL) provided by the Else-Kroeger-Fresenius Forschungskolleg (grant number: 2020_EKFK.15) at the Medical Center - University of Freiburg, Faculty of Medicine, University of Freiburg, Germany.

## Conflict of Interest

The authors declare that the research was conducted in the absence of any commercial or financial relationships that could be construed as a potential conflict of interest.
