# Supplementary material for: Case Report: Blurred Vision and Eruptive Nevi - Bilateral Diffuse Uveal Melanocytic Proliferation With Mucocutaneous Involvement in a Lung Cancer Patient
Source: Front Oncol. 2021 Apr 13;11:658407. doi: 10.3389/fonc.2021.658407 (PMC8076566; doi:10.3389/fonc.2021.658407)
Supplement: Supplementary file 1 [file DataSheet_1.docx]

Supplementary Material

# Supplementary methods

# Plasmapheresis

# Plasmapheresis was performed 11 times over a period of 3 weeks (5x week 1; 3x week 2 and 3). This regimen was adapted from the treatment of Guillain-Barré Syndrome and Multiple Sclerosis were early high frequency treatment is required to eliminate autoantibodies. Exchange volume was 2.5 L according to 40 ml / kg body weight (body weight was 63 kg). Human albumin and fresh frozen plasma (FFP) were used for plasma exchange with coagulation markers (Quick, PTT, AT3 and fibrinogen) being checked daily. Two plasma exchange devices were used: The Octo Nova® (Diamed, Köln, Germany) where plasma separation is done by a column (Plasmaflo OP-08W(L), Asahi Kasei Medical Co., Tokyo, Japan). The pore size was 0.3 µm and the column is made of polycarbonate. Heparin was used for anticoagulation (2500 IE initial bolus followed by 500 IE/h). The second device was the Spectra Optia® Apheresis System (TerumoBCT, Tokyo, Japan) were plasma separation works through centrifuge with 87% of mean plasma removal efficiency. Citrate was used for anti-coagulation in case of the Spectra Optia® Apheresis System. Both procedures were used with a similar frequency A Shaldon catheter was used to access circulation via the right V. jugularis interna.

# Melanocyte culture

# Human Epidermal Melanocytes (HEMn) isolated from lightly pigmented (LP) neonatal foreskin (HEMn-LP, Thermo Fisher Scientific, Waltham, Massachusetts, USA, Catalog Numbers C-002-5C) were thawed, cultivated and expanded in culture flasks (25 cm2) using culture medium 254 (Gibco/Thermo Fisher Scientific, Cat. No. M-254-500) supplemented with Human Melanocyte Growth Supplement (HMGS, Gibco/Thermo Fisher Scientific, Cat. no. S-002-5). Cells were then seeded in a 12 well plate with 15x103 cells per well in 2 ml medium comprising the following conditions (in duplicates): 1: medium + HMGS alone; 2: plasma from a healthy control (concentrations 0.5%, 1%, 5% and 10%); 3: patient plasma from first plasmapheresis (concentrations 0.5%, 1%, 5% and 10%); 4: patient plasma from second plasmapheresis (concentrations 0.5%, 1%, 5% and 10%); 5: patient plasma taken 4 months after last plasmapheresis (concentrations 0.5%, 1%, 5% and 10%). Daily medium changes were performed. On day seven images were taken and cells were removed from the culture plate using Accutase solution (Sigma-Aldrich, St. Louis, Missouri, Cat. No. A6964). To determine the cell number 20µl Trypan blue was mixed with 20µl cell suspension. Out of this 10 µl were taken for counting in a Neubauer chamber. Concentration per ml and total cell numbers were noted.
